# Assessing COVID-19 IgG levels among vaccinated and non-vaccinated individuals in Mthatha – South africa: A case-control approach

**DOI:** 10.1007/s13337-025-00942-w

**Published:** 2025-10-23

**Authors:** William Owusu, Gabriel Tchuente Kamsu, Eugene Jamot Ndebia

**Affiliations:** Department of Human Biology, Faculty of Medicine and Health Sciences, Walter Sisulu University, Nelson Mandela Drive, Mthatha, 5117 South Africa

**Keywords:** COVID-19, SARS-CoV-2 IgG, Saliva, Bio-Rad multiplex immunoassay

## Abstract

Faced with the emergence of new COVID-19 variants, vaccine hesitancy remains a major challenge in the global fight against the pandemic, particularly in rural areas. Understanding the immune response to vaccination is crucial for dispelling doubts and building public trust. By providing tangible evidence of vaccine effectiveness, this approach could not only alleviate skepticism but also contribute to preventing future epidemics. To evaluate and compare salivary SARS-CoV-2 IgG antibody levels between vaccinated and unvaccinated individuals in Mthatha, South Africa. A case-control study involved 183 participants aged 18 years and above, comprising 91 vaccinated and 92 unvaccinated individuals in Mthatha, South Africa. Saliva samples were collected and analyzed using Bio-Plex Pro Human IgG SARS-CoV-2 Assay to detect SARS-CoV-2 IgG antibodies. Data were processed using STATA/SE version 18.0. The results of this study demonstrate that participants vaccinated against COVID-19 exhibit significantly (*p* = 0.01) higher salivary IgG antibody levels (988 ± 104 pg/mL) compared to unvaccinated individuals (552 ± 83 pg/mL). No significant difference was observed between those vaccinated recently (975 ± 175 pg/mL) and those vaccinated more than two years ago (990 ± 215 pg/mL). Additionally, participants who received two doses showed notably higher antibody levels (1790 ± 445 pg/mL) compared to those who received a single dose (668 ± 75 pg/mL), with a significant difference (*p* = 0.004). In summary, vaccination is associated with higher salivary anti-SARS-CoV-2 IgG levels, influenced by vaccine type and number of doses. The detection of IgG in unvaccinated individuals and the lack of a defined protective correlate underscore the need for further studies on both humoral and cellular immunity to clarify the durability and magnitude of the immune response.

**Supplementary Information:**

The online version contains supplementary material available at 10.1007/s13337-025-00942-w.

## Introduction

The COVID-19 pandemic, caused by the severe acute respiratory syndrome related Coronavirus 2 (SARS-CoV-2), has significantly affected millions globally, with 492 million confirmed cases and over 6 million fatalities [1]. In Africa, the continent has reported over 12.2 million confirmed cases and 250,000 deaths, with South Africa accounting for 34% of these cases and 41% of the fatalities [2]. Within South Africa, the Eastern Cape Province has reported 185 995 cases and 11 673 deaths, with O.R. Tambo District contributing significantly to these figures [3]. The statistics emphasize the ongoing impact of COVID-19 on public health systems globally and regionally.

In late 2020, the Food and Drug Administration (FDA) approved the distribution and use of mRNA COVID-19 vaccines such as BNT162b2 (Pfizer) and messenger RNA (mRNA)−1273 (Moderna), set to be administered in two dosages [4]. This was followed by the approval of the Ad26.COV2 (Johnson & Johnson) vaccines are typically administered once [4], which has been proven effective in preventing COVID-19 [5]. Moreover, mRNA vaccines have been discovered to decrease the prevalence of asymptomatic illness and related infections significantly [6]. The BNT162b2 vaccine from Pfizer synthesizes SARS-CoV-2 spike proteins embedded on the target cells’ surface. This process enables the immune system to recognize these proteins, producing SARS-CoV-2-specific IgG antibodies. Consequently, an immune response is elicited, which plays a crucial role in preventing infection and mitigating the impact of the virus [7].

According to a survey conducted by Ipsos [8], 36% of South Africans were hesitant to receive the COVID-19 vaccine, highlighting that such reluctance poses a significant barrier to achieving herd immunity. This hesitation primarily stems from a lack of confidence in the safety, efficacy, and accessibility of vaccines, which threatens to worsen COVID-19 morbidity and mortality by allowing the virus to spread unchecked [9]. To address these concerns, strong evidence is needed to demonstrate that COVID-19 vaccinations effectively produce a robust protective immune response.

In this context, assessing SARS-CoV-2 specific IgG levels provides crucial insights into the immunity induced by vaccines. The choice of IgG is based on its abundance, extended half-life, sensitivity, and ability to retain memory of past infections [10]. Moreover, given that most studies rely on serum-based antibody assessments, using saliva appears as a less invasive and more practical approach to evaluate immune responses, particularly in community settings. Saliva was preferred over serum in this study because it is non-invasive. This choice avoids venipuncture, which is generally painful and often responsible for low participation rates, as well as the need for qualified phlebotomists, who are scarce in the area due to limited access to healthcare. In addition, saliva collection is simple, quick, painless and well accepted by participants, which promotes better adherence to research protocols. Saliva does not require such a strict cold chain as serum, which reduces the logistical constraints and costs associated with transporting and storing samples, a major advantage in rural areas with limited resources [11]. Furthermore, several studies have shown a good correlation between IgG levels for SARS-CoV-2 in saliva and those found in serum, allowing saliva to be used as a reliable alternative matrix for assessing humoral immunity [12]– [13]. Additionally, saliva offers a dependable method for detecting humoral responses in both those who are infected and those who are vaccinated, with recent comparisons revealing sensitivities ranging from 88 to 99% [12, 13]. Several studies have explored the use of saliva for SARS-CoV-2 diagnosis or detection in South Africa [14, 15], but none so far have focused on antibody surveillance especially in rural areas for IgG-based immunity profiling, which makes our approach particularly innovative in resource-limited settings. This study aims to bridge the gap between vaccine hesitancy and immunological evidence by quantifying and comparing IgG levels against SARS-CoV-2 in vaccinated and unvaccinated individuals in Mthatha. In other words, it aims to present solid evidence to alleviate vaccine hesitancy by demonstrating the improved immune responses observed in vaccinated individuals.

## Materials and methods

### Study design

This case-control study, carried out in Mthatha (coordinates: 31° 35‘20.148’‘ N and 28° 47’ 3.948‘’ E), a locality located in the O.R. Tambo district in the Eastern Cape province of South Africa, aimed to assess IgG levels against COVID-19 in the local population using saliva samples. Participants were recruited between April and June 2024 through convenience sampling in the Mthatha Central Business District, where consenting individuals were approached and informed about the study objectives. Convenience sampling in the Mthatha Central Business District leads to potential selection bias, over-representing accessible, urban, or health-conscious individuals and limiting generalizability to broader or more rural populations. Vaccinated participants were identified by their vaccination certificate. At the same time, those without access were asked to provide their RSA identification number and telephone number so they could retrieve their certificate via the South African government website (https://vaccine.certificate.health.gov.za/). Unvaccinated participants comprised participants who had never received a SARS-CoV-2 vaccine and had never undergone any treatment for SARS-CoV-2 infection. Participants were instructed not to eat, drink, smoke, or chew gum for 2 h before saliva collection to ensure reliable results. The sample size was calculated at 180 participants using the Bland guideline [16], divided into two groups of 90 (vaccinated and non-vaccinated). Inclusion criteria required participants to be adults (aged 18 or over) and consent to participate in the study, while exclusion criteria included people with chronic illnesses, pregnant women, and minors.

## Data collection and variables

A structured questionnaire was used to collect participant data, including demographic and vaccination-related variables. Continuous variables included age (in years) and SARS-CoV-2-specific IgG antibody levels (measured as Mean Fluorescence Intensity (MFI)) values from Bio-Plex multiplex assay on saliva samples. Categorical variables comprised gender (male, female, other), ethnicity (White, Black, Indian, other), vaccination status (vaccinated, unvaccinated), vaccine type (Pfizer-BioNTech, Johnson & Johnson, other), number of doses received (1, 2, or more), and vaccination period (categorized as 2020–2021 or 2022–2024 based on the date of the last dose).

## Saliva sample collection

Saliva collection was conducted from 10 AM to 1 PM, a few hours after the participants’ breakfast. Each participant was provided with sterile, ready-to-use 2 mL microcentrifuge tubes, each labeled with a unique identifier. Participants were instructed to rinse their mouths with water and wait 5 min before collecting saliva samples. They were requested to obtain at least 1 mL of saliva by drooling directly into the tubes. To preserve the integrity of the samples, participants were advised against forceful spitting, as this could lead to excessive bubbling and subsequently reduce the quality of the samples [17]. Upon achieving the required volume, participants were instructed to close the caps on the tubes securely. Finally, the tubes and the participants’ hands were wiped with tissue and sanitized to ensure cleanliness and safety.

Immediately after collection, saliva samples were kept in an ice-filled polystyrene cooler box to preserve the analytes [18]. After collecting saliva in the central business district of Mthatha, the samples were quickly taken to Walter Sisulu University’s tissue culture lab and kept in a freezer at −70 °C on the same day until analysis, following instructions from Salimetrics [18].

## Quantification of SARS-CoV-2 IgG antibodies with the Bio-Plex pro assay

The Bio-Plex Pro Human IgG SARS-CoV-2 N/RBD/S1/S2 4-Plex Panel (catalog #12014634, Bio-Rad Laboratories, Hercules, CA, USA) (www.bio-rad.com), was used to analyse IgG levels in saliva samples. This assay has been validated for saliva specimens by the manufacturer, demonstrating stability in MFI results under up to six freeze-thaw cycles and 6 days of refrigeration at 4 °C, with an optimal 2.5-fold dilution to maintain detectable antibody levels comparable to serum and plasma. This assay has been validated by the manufacturer for use with saliva samples, showing consistent MFI results after undergoing as many as six freeze-thaw cycles and being stored in refrigeration at 4 °C for 6 days, with a recommended dilution of 2.5-fold to keep antibody levels detectable at levels similar to those found in serum and plasma (www.bio-rad.com). Furthermore, studies that have undergone peer review have backed the use of Bio-Plex-based multiplex assays for detecting SARS-CoV-2 IgG in saliva, indicating a high level of performance with a sensitivity of 87.5% and specificity of 95.8% in convalescent samples, though noting waning of salivary IgG post-vaccination [19]. The kit includes reagents such as magnetic capture beads coupled to SARS-CoV-2 nucleocapsid (N), receptor-binding domain (RBD), spike 1 (S1), and spike 2 (S2) proteins; biotinylated anti-human IgG detection antibody (catalog #12014668); streptavidin-phycoerythrin (SA-PE) conjugate; positive and negative controls (catalog #12014774); serology sample diluent; detection antibody diluent; assay buffer; and wash buffer. All reagents were sourced from Bio-Rad and used according to manufacturer instructions.

For saliva sample preparation, the samples were thawed at room temperature and then diluted 2.5-fold with a sample diluent using a calibrated Eppendorf pipet. In each well of a 96-well plate, 50µL of 1x beads, 1x SA-PE, samples, controls, and 25µL of 1x detection antibody were added. The plate was then incubated at room temperature for 30 min and shaken at 850 rpm. After each addition, unbound components were removed by washing with 100µL of 1x wash buffer using a Bio-Plex Pro wash station (catalog #30034376, Bio-Rad Laboratories, Hercules, CA, USA). Data was collected using the Bio-Plex 200 Reader (catalog #171000201, Bio-Rad Laboratories, USA), which illuminates the fluorescent dyes in the beads with a red laser (635 nm). A green laser (532 nm) activates phycoerythrin, which emits a signal recorded by the photomultiplier tube (PMT). The Bio-Plex Manager software (version 6.2, Bio-Rad Laboratories, USA) processes the data as median fluorescence intensity (MFI), then expresses it in pg/mL following instructions by Bio-Rad (www.bio-rad.com). Although the cutoff values for the MFI of the Nucleocapsid protein (N) is > 450, the Receptor Binding Domain (RBD) is > 250, the Spike 1 subunit (S1) is > 250, and the Spike 2 subunit (S2) is > 750, this study did not retrieve specific cutoff values for each antigen. This may be due to data aggregation during post-processing or software settings combining the antigens (www.bio-rad.com).

### Statistical analysis

Data were entered and managed using Microsoft Excel 365, which was then presented as mean ± standard error of the mean (SEM) for continuous variables. while categorical variables were presented as percentages. The difference in antibody levels between vaccinated and unvaccinated participants was assessed using an unpaired Mann-Whitney U Test, as the Shapiro-Wilk test indicated non-normal distribution of our continuous data. P values below or equal to 0.05 (*P* ≤ 0.05) were considered statistically significant. Similar non-parametric tests were applied for subgroup comparisons. Version 18.0 of STATA/SE was used for statistical analyses.

### Ethical considerations

This study was approved by Walter Sisulu University’s ethics committee (reference number 023/2023). In this study, participants’ names were not used. They were given codes, and the measurements taken were confidential. Consent forms and participant data are stored in a lockable cabinet where only the research team can access them. Participants enrolled in the study voluntarily and were free to leave at any time if they felt violated.

##  Results

### Demographic parameters of studied participants

Table 1 shows the demographic parameters of the 183 participants recruited in this study. Analysis of this table reveals that the mean age among the participants was 26 years. Among the participants, 91 (49.7%) had received the vaccination, while 92 (50.2%) had not. Of the 91 individuals who received the vaccination, 49.5% were men and 50.5% were women. Among the unvaccinated group (*N* = 92), gender distribution between the two groups was identical at 50% each. Most vaccinated individuals received the Johnson & Johnson vaccine (66%), followed by the Pfizer vaccine (30.8%). A small percentage of 1.1% opted for the Moderna vaccine, while 2.2% were unsure of their vaccination type. Most vaccinated individuals received two doses (70.3%), and 27% received a single dose, while 2.2% were unsure of their vaccination dosage. Significant vaccinations occurred in 2021, with 47% of individuals vaccinated during that year. However, there was a decline to 29% in 2022 and a further decrease to 9% in 2023. Moreover, a small proportion (5%) were vaccinated during the peak of COVID-19 in 2020. Additionally, 13 individuals in the vaccinated group had IgG levels below the average concentration of unvaccinated participants. Among the 13 vaccinated participants whose IgG levels were below the mean observed in unvaccinated individuals, 12 had received the single-dose Johnson & Johnson vaccine. The thirteenth participant had received a Pfizer vaccine and may represent a potential non-responder. However, although no unvaccinated participants had zero IgG concentrations, 15 participants had IgG values below or equal to 50 pg/mL.

**Table 1:** Demographic parameters of studied participants, including age, gender, vaccine type, number of doses, and vaccination year

Variables	Categories	Vaccinated group (*N* = 91)	Unvaccinated group (*N* = 92)	Overall (*N* = 183)
		Frequency (%)	Frequency (%)	Frequency (%)
**Gender**	Male Female	45 (49.5) 46 (50.5)	46 (50) 46 (50)	91 (49.7) 92 (50.3)
**Vaccine type**	J&J Pfizer Moderna Unknown	60 (66) 28 (30.8) 1 (1.1) 2 (2.2)	N/A	60 (32.8) 28 (15.3) 1 (0.5) 2 (1.1)
**Year of vaccination**	2023 2022 2021 2020 Unknown	9 (9.9) 29 (31.9) 47 (51.6) 5 (5.5) 1 (1.1)	N/A	9 (4.9) 29 (15.8) 47 (25.7) 5 (2.7) 1 (0.5)
**Dosage**	Once Twice Unknown	64 (70.3) 25 (27.5) 2 (2.2)	N/A	64 (35) 25 (13.7) 2 (1.1)
**Mean Age (Years)**		26 ± 1	24 ± 0.5	25 ± 1

J&J: Johnson & Johnson; N/A: Not Applicable. Percentages calculated separately for vaccinated and unvaccinated groups

## Differences in SARS-CoV-2 IgG levels between vaccinated and unvaccinated participants

**Fig. 1:**
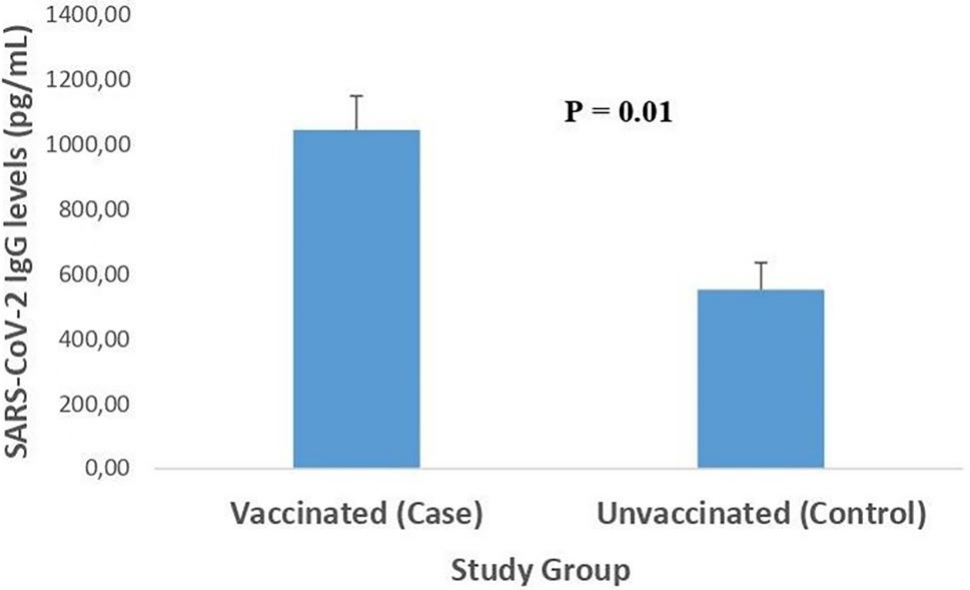
Differences in salivary SARS-coV-2 levels between vaccinated and unvaccinated participants. Data are expressed as mean $\pm$ SEM. P value was determined using the Mann- Whitney U test (p <0.01)

Figure 1 reveals a substantial difference in the IgG antibody levels between vaccinated and unvaccinated individuals in response to SARS-CoV-2. The mean level of SARS-CoV-2 IgG antibodies in the vaccinated group was 988 ± 104 pg/mL, which is significantly higher than the IgG level observed in unvaccinated individuals (552 ± 83 pg/mL).

### Impact of time after vaccination on SARS-CoV-2 IgG levels in vaccinated individuals

**Fig. 2:**
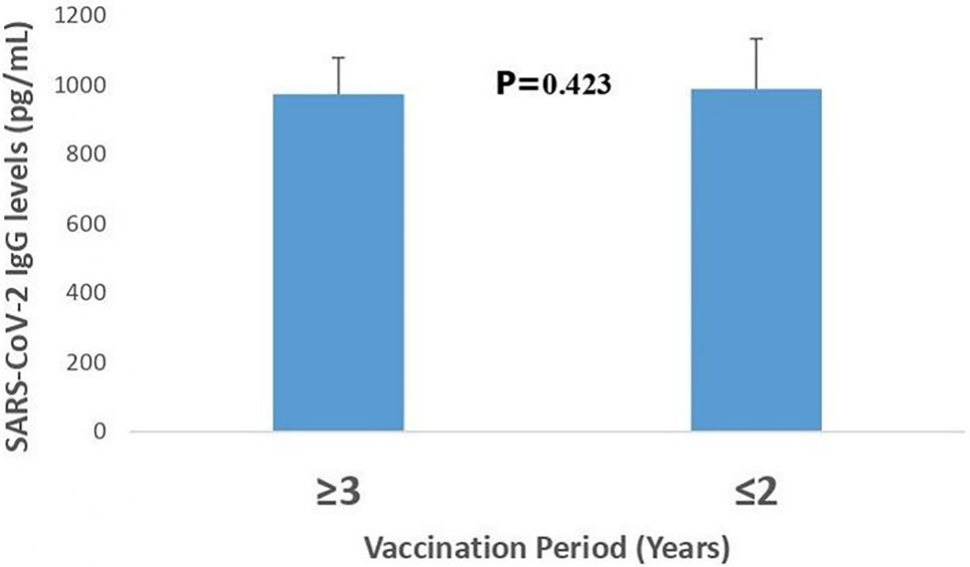
"Comparison of salivary SARS-coV-2 IgG antibody levels(pg/ml) among participants, among grouped by time since vaccination ($\leq$2 years vs.$\geq$ 3 years). Values mean $\pm$ SEM, analyzed using, Mann Whitney U test

Figure 2 compares SARS-CoV-2 IgG levels in vaccinated individuals over time. This figure illustrates the lack of a significant difference (*P* = 0.423) in mean anti-SARS-CoV-2 IgG antibody levels between participants vaccinated between 2020 and 2021 (≥ 3 Years) and those vaccinated between 2022 and 2023 (≤ 2 Years), with antibody concentrations of 975 ± 105 pg/mL and 990 ± 145 pg/mL, respectively.

### SARS-CoV-2 IgG response in Pfizer and J&J vaccinated participants

**Fig. 3:**
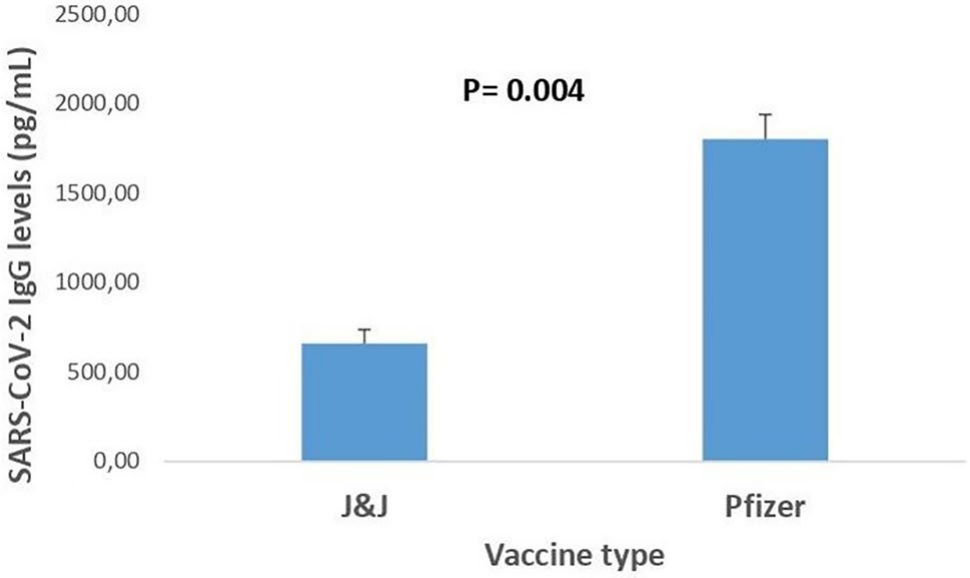
Comparison of salivary SARS-coV-2 IgG antibody levels(pg/ml) among between Pfizer (two-dose) and Johnson and Johnson (single-dose) vaccine receipts. Data re shown as mean$\pm$ SEM. A statistically significant difference (p=0.004, Mann Whitney U test) is included

Figure 3 compares the mean SARS-CoV-2 IgG levels (pg/mL) between individuals vaccinated with the Pfizer and J&J vaccines as well as the number of doses. The graph shows that participants who were administered the Pfizer vaccine exhibited significantly higher SARS-CoV-2 antibody levels, averaging 1648 ± 138 pg/mL. In contrast, those who received the J&J vaccine displayed lower antibody levels, averaging 659 ± 78 pg/mL. The difference in antibody levels between the two vaccines was statistically significant, with a p-value of 0.004. This figure also shows that the anti-SARS-CoV-2 antibody levels of participants who received two doses of vaccine (Pfizer) were significantly higher, while those who received a single dose (J&J) were lower.

## Discussion

This study demonstrates the feasibility, accessibility, and practicality of detecting SARS-CoV-2 antibodies via saliva samples, a non-invasive alternative to serum that promotes equity in rural areas like Mthatha [11]. Our findings reveal significantly higher SARS-CoV-2-specific IgG levels in vaccinated versus unvaccinated participants, underscoring vaccination’s role in enhancing humoral immunity and reducing severe disease risk. There were similar findings in the USA, Libya, and China populations [20–24]. Aligning with our results, a Brazilian study reported significantly higher salivary IgG in vaccinated healthcare workers compared to unvaccinated controls [25]. In India, while total salivary antibodies were higher in vaccinated young adults (Covaxin or Covishield), IgG levels showed no statistically significant difference from unvaccinated individuals, potentially influenced by factors like age or vaccine type, contrasting with our observed disparity [26]. US-based research on mRNA vaccines (Pfizer/Moderna) demonstrated detectable salivary IgG persisting post-vaccination, mirroring our findings, though some studies noted shorter-lived responses in saliva compared to serum [27]– [28].

The detectability of SARS-CoV-2 antibodies in unvaccinated individuals also suggests previous natural infections or cross-reactivity with other coronaviruses. Natural infection also induces protective immune responses, but hybrid immunity (vaccination plus infection) provides superior durability, breadth, and cross-variant defense through amplified T cell and memory B cell responses, which provide protection against severe illnesses for at least 6 to 12 months following exposure [19, 29, 30]. However, potential confounding factors such as previous COVID-19 infections or asymptomatic exposures could influence observed IgG levels. Furthermore, an unvaccinated participant who is asymptomatically infected with SARS-CoV-2 may exhibit elevated antibody titres. This elevation could potentially distort the comparisons between different study groups [31]. Furthermore, factors such as viral load and illness severity further modulate antibody persistence, inflating baseline immunity in study cohorts [28]. Cross-reactivity with other pathogens (e.g., endemic coronaviruses or dengue virus) may also contribute to these distortions, as supported by studies demonstrating antibody-binding overlaps that can lead to false positives or altered responses, though cross-neutralization is often rare [32]. To mitigate these biases, in the absence of a clearly established protective correlate, future research needs to incorporate participant history and both humoral and cellular immunity to better understand the durability and extent of the immune response.

The Pfizer-BioNTech vaccine, which utilizes messenger RNA (mRNA) technology, has induce higher IgG titers and cellular immunity compared to the Johnson & Johnson vaccine, which implements a viral vector methodology. This aligns with Brazilian findings where Pfizer boosters after CoronaVac enhanced salivary IgG persistence compared to inactivated vaccines alone [33]. The results observed can be linked to the synthetic version of the complete spike glycoprotein (S) of the SARS-CoV-2 virus present in the mRNA vaccine. The spike protein is a crucial antigen that helps neutralize antibodies necessary to stop the virus from entering host cells and replicating further [7, 33]. Importantly, the research emphasizes that multi-dose vaccines not only generate more robust immediate antibody responses but may also support long-term immunity by improving the activation of memory B cells [34]. Nevertheless, further large-scale studies in South Africa must confirm the discrepancies between single-dose and multi-dose vaccine regimens, particularly in diverse populations with varying genetic and environmental factors.

No significant IgG differences emerged between participants vaccinated in 2020–2021 versus 2022–2024. These observations suggest that, in our cohort, salivary IgG remains relatively stable over time, which is consistent with the robustness of the humoral response induced by vaccination. However, this result contrasts with the work of [35], who reported a considerable decrease in anti-SARS-CoV-2 IgG levels in saliva 180 days after vaccination in London (UK), highlighting that the persistence of salivary IgG may vary depending on the geographical context, the populations studied and the types of vaccines used. This suggests sustained immunity via memory B and T cells, which enable rapid recall and affinity-matured responses despite waning circulating antibodies [34, 36–38]. Comparable longevity was observed in US and European studies, with salivary IgG detectable up to 9–12 months post-vaccination or infection [39], though some reported faster decline in mucosal responses [40]. This implies that booster strategies could be tailored based on individual immune monitoring rather than fixed schedules, potentially optimizing vaccine distribution in underserved regions. However, as SARS-CoV-2 evolves (e.g., Omicron variants), monovalent vaccines may require updates or polyvalent formulations targeting conserved epitopes for broader, longer-term protection [41]. In rural South Africa, these results advocate for expanded saliva-based surveillance to guide targeted boosters, hybrid immunity promotion through combined vaccination and infection management, and investment in universal vaccines to enhance community resilience against emerging variants, as evidenced by ongoing Phase I clinical trials and new platforms in 2025 [42].

### Limitations

This study provides valuable information regarding SARS-CoV-2-specific IgG levels in saliva from a cohort in the Mthatha Central Business District of South Africa. However, notable limitations include the lack of reported rates of unvaccinated individuals who do not show IgG positivity, which complicates the evaluation of baseline seroprevalence and undetected cases; there is also an absence of defined cut-offs for IgG positivity, as the focus was solely on comparing mean levels. Future research could establish validated thresholds. Furthermore, the cross-sectional nature of our research, which captures data at a single point in time without longitudinal follow-up, limits our ability to interpret dynamic changes in immune response, such as waning or boosting of SARS-CoV-2 IgG levels over time. The comparative analysis aimed to highlight differences in antibody responses post-vaccination versus natural exposure. Future studies could incorporate measures to address these limitations and build on the initial thought. The potential for recall bias is a notable concern, particularly with our exclusion criterion for underlying diseases, which could elevate IgG levels. Participants may not fully disclose their health status intentionally or unintentionally, potentially skewing results. Furthermore, convenience sampling in the Mthatha Central Business District introduces selection bias, as it may over-represent accessible, urban, or health-conscious individuals, limiting generalizability to broader or more rural populations. While our data support the durability of memory-mediated immunity against severe disease in the studied cohorts, we concur that long-term protection from monovalent vaccines alone is unlikely to persist indefinitely against evolving variants. Future research should prioritise stricter criteria, randomised sampling, and evaluations of next-generation polyvalent vaccines, as well as the measurement of IgA in the buccal mucosa, in diverse South African populations to enhance resilience and equity in global immunity.

## Conclusion

Overall, this study demonstrates that South African populations who received the COVID-19 vaccine could potentially benefit from long-term immunological memory against SARS-CoV-2. Furthermore, it highlights the potential of saliva-based tests, providing an accessible and effective solution for public health monitoring and supporting vaccination campaigns. By proving the prolonged immunological memory of vaccines and offering an innovative diagnostic alternative, this research marks a significant step in combating vaccine hesitancy and paves the way for new public health strategies in South Africa.

## Supplementary Information

Below is the link to the electronic supplementary material.

Supplementary Material 1

## Data Availability

Due to legal restrictions imposed by the South African government under the “Personal Information Protection Act”, data cannot be made public.

## Abbreviations

FDA: Food and Drug Administration
Ig: Immunoglobulin
J&J: Johnson and Johnson
RNA: Ribonucleic Acid
SA: PE–Streptavidin–Phycoeythrin
SARS: CoV–2–Severe acute respiratory syndrome coronavirus 2
SEM: Standard error of the mean
WHO: World Health Organisation

## Declarations

### Statement

We confirm that this manuscript has not been previously published and is not currently under consideration for publication elsewhere.
