# Supplementary material for: Assessing COVID-19 IgG levels among vaccinated and non-vaccinated individuals in Mthatha – South africa: A case-control approach
Source: Virusdisease. 2025 Oct 23;36(3):475–83. doi: 10.1007/s13337-025-00942-w (PMC12634943; doi:10.1007/s13337-025-00942-w)
Supplement: Supplementary file 1 — Supplementary Material 1 [file 13337_2025_942_MOESM1_ESM.docx]

**Assessing COVID-19 IgG levels among vaccinated and non-vaccinated individuals in Mthatha – South Africa: A case-control approach**

William Owusu; Gabriel Tchuente Kamsu; Eugene Jamot Ndebia*

Department of Human Biology, Walter Sisulu University, Nelson Mandela Drive, Mthatha, 5117, South Africa

*Corresponding author: Eugene Jamot Ndebia ([endebia@wsu.ac.za](mailto:endebia@wsu.ac.za))

Authors informations :

## **William Owusu**: E-mail: [wowusu@wsu.ac.za](mailto:wowusu@wsu.ac.za); ORCID: <https://orcid.org/0000-0003-4083-8871>

## **Gabriel Tchuente Kamsu**: E-mail: [gkamsu-tchuente@wsu.ac.za](mailto:gkamsu-tchuente@wsu.ac.za); ORCID: <https://orcid.org/0000-0001-5641-5916>

## **Eugene Jamot Ndebia**: E-mail: [endebia@wsu.ac.za](mailto:endebia@wsu.ac.za) ; ORCID: <https://orcid.org/0000-0002-5840-0715>

**Abbreviations**

FDA – Food and Drug Administration

Ig – Immunoglobulin

J&J – Johnson and Johnson

RNA – Ribonucleic Acid

SA-PE – Streptavidin-Phycoeythrin

SARS-CoV-2 – severe acute respiratory syndrome coronavirus 2

SEM – Standard error of the mean

WHO – World Health Organisation

**Acknowledgments**: The authors would like to express their sincere gratitude to all the participants involved in this study and to Walter Sisulu University, particularly the Department of Human Biology, for providing the necessary facilities, tools, and equipment to complete this research project.

**Funding:** This study was supported by the Chemical Industries Education and Training Authority (CHIETA) and the National Research Foundation (NRF), fundings attributed to Prof. Eugene Jamot Ndebia and William Owusu, respectively.

**Data availability:** Due to legal restrictions imposed by the South African government under the “Personal Information Protection Act”, data cannot be made public.

**Declarations**

**IRB approval status:** This study was conducted in accordance with the Declaration of Helsinki and approved by the Institutional Ethics Committee of the Faculty of Medicine and Health Sciences, Walter Sisulu University with approval number 023/2023.

**Conflict of interest:** The authors have no relevant affiliations or financial involvement with any organization or entity with a financial interest in or financial conflict with the subject matter or materials discussed in the manuscript.

**Statement:** We confirm that this manuscript has not been previously published and is not currently under consideration for publication elsewhere.
